# Jasmonate-Responsive Transcription Factors NnWRKY70a and NnWRKY70b Positively Regulate Benzylisoquinoline Alkaloid Biosynthesis in Lotus (*Nelumbo nucifera*)

**DOI:** 10.3389/fpls.2022.862915

**Published:** 2022-06-15

**Authors:** Jing Li, Yi Li, Mingjing Dang, Shang Li, Simeng Chen, Ruizhen Liu, Zeyu Zhang, Guoqian Li, Minghua Zhang, Dong Yang, Mei Yang, Yanling Liu, Daike Tian, Xianbao Deng

**Affiliations:** ^1^School of Chemistry, Chemical Engineering and Life Sciences, Wuhan University of Technology, Wuhan, China; ^2^Aquatic Plant Research Center, Wuhan Botanical Garden, Chinese Academy of Sciences, Wuhan, China; ^3^Shanghai Key Laboratory of Plant Functional Genomics and Resources, Shanghai Chenshan Botanical Garden, Shanghai, China

**Keywords:** NnWRKY70, transcriptional regulation, lotus, benzylisoquinoline alkaloid biosynthesis, secondary metabolite

## Abstract

Lotus (*Nelumbo nucifera*) is a large aquatic plant that accumulates pharmacologically significant benzylisoquinoline alkaloids (BIAs). However, little is known about their biosynthesis and regulation. Here, we show that the two group III WRKY transcription factors (TFs), NnWRKY70a and NnWRKY70b, positively regulate the BIA biosynthesis in lotus. Both NnWRKY70s are jasmonic acid (JA) responsive, with their expression profiles highly correlated to the BIA concentration and BIA pathway gene expression. A dual-luciferase assay showed that NnWRKY70a could transactivate the *NnTYDC* promoter, whereas NnWRKY70b could activate promoters of the three BIA structural genes, including *NnTYDC, NnCYP80G*, and *Nn7OMT*. In addition, the transient overexpression of *NnWRKY70a* and *NnWRKY70b* in lotus petals significantly elevated the BIA alkaloid concentrations. Notably, NnWRKY70b seems to be a stronger BIA biosynthesis regulator, because it dramatically induced more BIA structural gene expressions and BIA accumulation than NnWRKY70a. A yeast two-hybrid assay further revealed that NnWRKY70b physically interacted with NnJAZ1 and two other group III WRKY TFs (NnWRKY53b and NnWRKY70a), suggesting that it may cooperate with the other group III WRKYs to adjust the lotus BIA biosynthesis via the JA-signaling pathway. To illustrate the mechanism underlying NnWRKY70b-mediated BIA regulation in the lotus, a simplified model is proposed. Our study provides useful insights into the regulatory roles of WRKY TFs in the biosynthesis of secondary metabolites.

## Introduction

Benzylisoquinoline alkaloids (BIA) are a diverse group of nitrogen-containing secondary metabolites in plants, with over 2,500 known structures to date (Facchini, 2001). Unlike other secondary metabolites that are found in most of the higher plants, BIAs only occur in limited plant families, such as Magnoliaceae, Ranunculaceae, Papaveraceae, and Berberidaceae, and are mostly pharmacologically significant. The typical medicinal BIAs include morphine and codeine (narcotic analgesics), sanguinarine and berberine (anti-microbial agents), tubocurarine and papaverine (muscle relaxants), and noscapine (cough suppressant and anti-cancer agent) (Hagel and Facchini, 2013).

**Figure 1:**
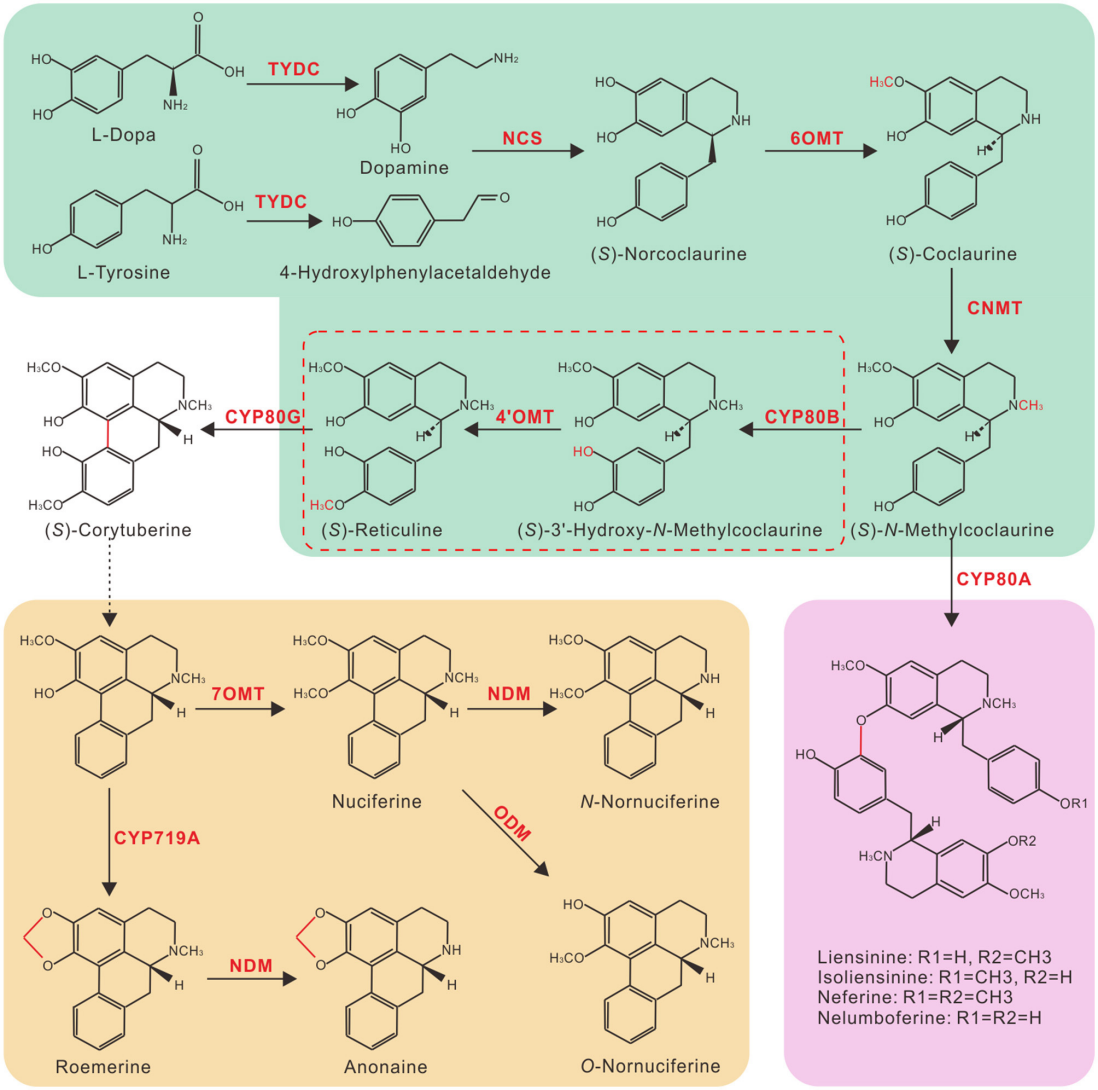
Putative benzylisoquinoline alkaloid biosynthesis (BIA) pathway in lotus. Branch pathways are shown in colored boxes: green, common steps to (*S*)-Reticuline; brown, aporphine alkaloids in lotus leaves; and purple, bis-benzylisoquinoline alkaloids in lotus embryos. Solid arrows indicate single-step reactions, dotted arrow indicates multiple steps, and the dotted box indicates possible redundant enzymatic steps in lotus. TYDC, tyrosine/DOPA decarboxylase; NCS, (*S*)-norcoclaurine synthase; 6OMT, (*S*)-norcoclaurine 6-*O*-methyltransferase; CNMT, (*S*)-coclaurine *N*-methyltransferase; CYP80B, (*S*)-*N-*methylcoclaurine 3′-hydroxylase; 4′OMT, (*S*)-3′-hydroxy-*N*-methylcoclaurine 4′-*O*-methyltransferase; NDM, *N*-demethylase; ODM, *O*- demethylase.

Despite their high-structural diversity, the biosynthesis of all BIAs in plants is conserved to a single L-tyrosine substrate origin. Tyrosine/DOPA decarboxylase (TYDC) converts L-tyrosine into dopamine and 4-hydroxyphenylacetaldehyde, which are subsequently condensed by (*S*)-norcoclaurine synthase (NCS) to yield (*S*)-norcoclaurine, the common precursor of all plant BIAs (Figure 1) (Facchini and De Luca, 1994; Maldonado-Mendoza et al., 1996; Samanani et al., 2004; Minami et al., 2007). Four additional enzymatic steps, catalyzed by (*S*)-norcoclaurine 6-*O*-methyltransferase (6OMT), (*S*)-coclaurine *N*-methyltransferase (CNMT), (*S*)-*N-*methylcoclaurine 3′-hydroxylase (CYP80B), and (*S*)-3′-hydroxy-*N*-methylcoclaurine 4′-*O*-methyltransferase (4′OMT), convert (*S*)-norcoclaurine to (*S*)-reticuline, a common intermediate branch point of most BIAs (Sato et al., 1994; Pauli and Kutchan, 1998; Morishige et al., 2000; Samanani et al., 2004). In the past decades, efforts have been devoted to elucidate the biosynthetic pathways of morphine, berberine, and sanguinarine because of their pharmacological significance. As a result, nearly all enzymes involved in the biosynthesis of these BIAs have been characterized, and *Papaver somniferum, Coptis japonica*, and *Eschscholzia californica* became model species for the study of BIA biosynthesis.

In plants, the biosynthesis and proper accumulation of BIAs are strictly controlled in a spatial and temporal manner, and may be influenced by numerous internal and external signals. Jasmonic acid (JA) and its derivatives are plant stress hormones that regulate various stress responses, such as microbial infections, herbivores, wounding, and UV-irradiation (Zhou and Memelink, 2016). The external application of JAs can induce the biosynthesis of most secondary metabolites by activating JA-responsive transcription factors (TFs), including AP2/ERFs, bHLHs, MYBs, and WRKYs (Patra et al., 2013; Wasternack and Strnad, 2019). These TFs in turn bind to specific *cis*-regulatory sequences of the biosynthetic gene promoters, leading to their transcriptional activation. In contrast, jasmonate ZIM-domain proteins (JAZ) are key negative regulators of JA signaling by binding to JA-responsive TFs and repressing their transcription. Exogenous JA treatment induces JAZ protein degradation, which sets the JAZ-repressed TFs free and activates the JA-responsive metabolic pathways (Santner and Estelle, 2007; Song et al., 2014; Wasternack and Strnad, 2019).

WRKY-domain-containing genes form one of the largest TF families in plants and play essential roles in the plant JA-signaling cascade (Eulgem et al., 2000; Rushton et al., 2010; Chen et al., 2018). The polypeptide sequences of these TFs characteristically comprise a WRKY DNA binding domain of approximately 60 amino acids, with one or two highly conserved WRKYGQK heptapeptides in the N-terminals and a C2H2 or C2HC zinc finger motif in the C-terminals (Eulgem et al., 2000; Li et al., 2019). Studies have shown that WRKY TFs, especially members of group III, are involved in the regulation of secondary metabolite biosynthesis in various medicinal plants. For example, CrWRKY1, a group III WRKY protein of *Catharanthus roseus*, positively regulates the monoterpenoid indole alkaloid (MIA) biosynthesis through binding and activating the *CrTDC* gene in the MIA biosynthetic pathway (Suttipanta et al., 2011). CjWRKY1, a *Coptis japonica* WRKY in the IIc group, has been reported to be JA responsive and specifically bound to the W-boxes in the structural gene promoters to regulate the berberine biosynthesis (Kato et al., 2007; Yamada and Sato, 2016). More recently, three group III WRKYs in *Ophiorrhiza pumila* were shown to regulate the biosynthesis of the anticancer drug camptothecin. Of them, OpWRKY1 represses the expression of *OpCPR*, and negatively regulates camptothecin accumulation (Xu et al., 2020), whereas both OpWRKY2 and OpWRKY3 could activate the camptothecin pathway gene expression and positively regulate the camptothecin biosynthesis (Wang et al., 2019; Hao et al., 2021).

Lotus (*Nelumbo nucifera*) is an aquatic plant species widely cultivated in Asian countries (Deng et al., 2022). In addition to their attractive flowers and nutritious rhizome and seeds, lotuses are rich in valuable medicinal BIAs, including nuciferine, *N*-nornuciferine, *O*-nornuciferine, roemerine, and anonaine in lotus leaves, as well as Liensinine, Isoliensinine, and Neferine in lotus embryos (Deng et al., 2016, 2018; Lin et al., 2019). Lotus contains 65 WRKY encoding genes, 34 of which are JA responsive and are deemed to be potential BIA biosynthesis regulators (Li et al., 2019). Previously, we have shown that two JA-responsive lotus WRKYs, NnWRKY40a and NnWRKY40b of the IIa group, could activate the promoters of two BIA biosynthetic genes (Li et al., 2019). Here, we present an extensive functional evaluation of two other lotus group III WRKY proteins: NnWRKY70a and NnWRKY70b. Our results showed that both NnWRKY70s positively regulate the BIA accumulation in the lotus through the transcriptional modulation of structural gene expressions.

## Materials and Methods

### Plant Material, JA Treatment, and Alkaloid Quantification

The lotus variety “Qiuxing” was rhizome propagated in pots of 40 cm diameter and 40 cm height in late April. All pots were set outdoor on a flat ground at the Wuhan Botanical Garden of the Chinese Academy of Sciences (Wuhan, Hubei province, China). Routine water and fertilization management were applied to all the plants during the growth season. *Nicotiana benthamiana* plants were cultivated in the growth room under controlled conditions: day/night temperature, 24/22°C; day/night length, 16/8 h; light intensity, 250 μmol m^−2^s^−1^; and relative humidity, 60%.

For JA treatment, methyl jasmonate (MeJA, 100 μM) was exogenously applied on lotus leaves at developmental stage 3 (S3) (Deng et al., 2016) and then wrapped with transparent plastic bags. A final concentration of 100 μM MeJA was prepared by first dissolving 11.2 μL MeJA (4.4 M) in 488.8 μL pure ethanol to make a 100 mM (1000× ) MeJA stock solution, which was then diluted with deionized water to the desired concentration. Leaf samples were collected at 0, 3, 6, and 24 h time intervals after MeJA treatment. For a tissue-specific expression analysis, eight lotus organ samples, including, root, rhizome, leaf, petal, embryo, seedpod, petiole, and stamen, as well as leaves at seven developmental stages (S1–S7) were harvested in July. All the samples were frozen immediately in liquid nitrogen and then stored at −80°C until use. The BIA alkaloids in the lotus were extracted and quantified as previously described (Deng et al., 2016).

### Bioinformatic and Phylogenetic Analysis

The protein sequences of all WRKYs previously reported to be involved in secondary metabolism were obtained from the National Center for Biotechnology Information (NCBI) GenBank. The genomic and coding sequences of *AtWRKY70* were retrieved from the TAIR database (https://www.arabidopsis.org/). The sequences of lotus genes were PCR amplified from the lotus variety “Qiuxing” and have been deposited in the GenBank with the following accession numbers: *NnWRKY70a*, OL469000; *NnWRKY70b*, OL469001; *NnWRKY53b*, OL468999; and *NnJAZ1*, OL469002. The full-length protein sequences were aligned with MUSCLE (Edgar, 2004), followed by a phylogenetic analysis in MEGA7.0 (Kumar et al., 2016) with a neighbor-joining method and 1,000 bootstrap replicates. The tree was finally viewed and modified with FigTree V1.4.2.

### Quantitative Real-Time PCR

Total RNA was extracted using the RNAprep Pure Plant Kit (Tiangen Biotec, Beijing, China), and cDNA was synthesized with the cDNA Synthesis SuperMix (TransGen, Beijing, China). The quantitative real-time PCR was carried out using SYBR^®^ Premix Ex Taq™ II (Takara, Dalian, China) on a StepOnePlus^TM^ Real-Time PCR System (Applied Biosystems, Foster City, CA, USA). The 2^−ΔΔCt^ method was used to calculate the relative gene expression (Pfaffl, 2001), and the *NnACTIN* gene was used as an internal reference for the normalization of gene expression levels (Gu et al., 2013). The primers used for real-time PCR are listed in Supplementary Table S1.

### Subcellular Localization Assay

The full-length coding sequences of *NnWRKY70a* and *NnWRKY70b* without the stop codon were amplified and inserted into the pMDC83 vector to generate CaMV 35S:NnWRKY70a-GFP and 35S:NnWRKY70b-GFP constructs. The empty pMDC83-GFP vector was used as a control. The recombinant constructs were transformed into the *Agrobacterium* GV3101 strain. Agro-infiltration was conducted on fully expanded leaves of 5–6-weeks-old *N. benthamiana* plants. The subcellular localization of fluorescent protein fusions was checked 2 days after the inoculation. A 5 μg/mL DAPI nuclear marker was infiltrated into the same area of agro-infiltration 30 min before sampling. The images were taken with the Zeiss Confocal Fluorescence Microscope (LSM710 Meta, Carl Zeiss) as previously reported (Li et al., 2019). The primers used for cloning subcellular localization vectors are listed in Supplementary Table S1.

### Dual-Luciferase Reporter Assay

The promoter regions spanning about 1.5–2 kb of *NnNCS1, NnTYDC1, NnCYP80G*, and *Nn7OMT* genes were PCR amplified and inserted into the dual-luciferase (LUC) reporter gene expression vector pGreen 0800-LUC to form *NnNCS1*pro::LUC, *NnTYDC1*pro::LUC, *NnCYP80G*pro::LUC, and *Nn7OMT*pro::LUC constructs. The complete coding sequence (CDS) of *NnWRKY70a* and *NnWRKY70b* genes were cloned into the pSAK277 vector under the 35S promoter to form the TF expression vectors. *Agrobacterium* transformation, agro-infiltration, and the measurements of Firefly luciferase (F-Luc) and Renilla luciferase (R-Luc) activities were performed as previously described (Deng et al., 2018). The primers used in the dual-luciferase reporter assay are listed in Supplementary Table S1.

### Yeast Hybrid Assays

The yeast one-hybrid assay was conducted according to the instructions of Matchmaker^®^ Gold Yeast One-Hybrid Library Screening System User Manual (Clonteck, USA). The promoter sequences of approximately 1.5-kb upstream of the BIA pathway gene start codon were PCR amplified and cloned into pAbAi vectors to generate bait-reporter yeast strains, while whole coding sequences of *NnWRKY70a* and *NnWRKY70b* were cloned into the pGADT7 vector to generate pGADT7-TF constructs. The minimal inhibitory concentration of Aureobasidin A (100 ng/mL) was determined with different bait-reporter yeast strains transformed with empty pGADT7 AD vectors. To evaluate whether NnWRKY70a and NnWRKY70b interact with BIA pathway gene promoters, pGADT7-TF plasmids were transformed into Y1HGold bait strains carrying different promoters and cultured on SD/-Leu containing 100 ng/mL AbA. Y1HGold bait strains transformed with empty pGADT7 were set as negative controls, while Y1HGold (p53-AbAi) transformed with pGADT7-p53 was set as a positive control. To check the specific binding of NnWRKY70a and NnWRKY70b to the W-box *cis*-elements, the promoter mutants were generated with overlap extension PCR to remove all W-box *cis*-elements.

For the yeast two-hybrid library screening, a lotus “Mate & Plate” library was constructed in Y187 yeast strain according to the “Make Your own Mate & Plate Library System User Manual” (Clonteck, USA). cDNA for the library was generated from a tissue mixture including leaves, petals, and embryos of the lotus variety “China Antique”. The library screening and yeast two-hybrid assays (Y2H) were conducted as described in the “Matchmaker Gold Yeast Two-Hybrid System User Manual” (Clonteck, USA). Truncated *NnWRKY70b* coding sequence harboring the WRKY domain was PCR amplified and cloned into the pGBKT7 (BD) vector as a bait to avoid autoactivation, while the full-length CDS of *NnWRKY70a, NnWRKY70b, NnWRKY53b*, and *NnJAZ1* were cloned into the pGADT7(AD) vector as a prey. Combinations of the different prey vectors and BD-NnWRKY70b bait were co-transformed into the Y2H Gold yeast strain. Meanwhile, the combinations of BD-53 + AD-T and BD-Lam + AD-T were co-transformed as positive and negative controls, respectively. The yeast cells were cultured on DDO (SD/-Leu/-Trp) selective medium and on TDO (SD/-Leu/-Trp/-His) and QDO (SD/-Leu/-Trp/-His/-Ade) media for interaction assays. The primers used for cloning Y1H and Y2H vectors are listed in Supplementary Table S1.

### Transient Overexpression of *NnWRKY70s* in Lotus Petals

Transient overexpression of *NnWRKY70s* was conducted in the lotus petals of the “Qiuxing” variety 2 days before blooming. *Agrobacterium* GV3101 strain carrying the pMDC83:NnWRKY70a and pMDC83:NnWRKY70b vectors were infiltrated on the lower side of petals at OD600 = 0.5, using a 1 mL needleless syringe. The lotus petals were sampled 2 days after infiltration for subsequent gene expression analysis and determination of alkaloid content.

### Bimolecular Fluorescence Complementation (BiFC) Assay

BiFC assay was conducted with a 2-in-1 system based on the splitting enhanced yellow fluorescent protein (EYFP) as previously described (Grefen and Blatt, 2012). Coding DNA sequences of two independent genes were gateway cloned into two different expression cassettes on the pBiFCt-2in1-CN vector. To construct the NnWRKY70b:nYFP fusion protein expression cassette, a termination code of NnWRKY70b was dropped out. *Agrobacterium tumefaciens* strain GV3101 carrying different constructs was infiltrated in *N. benthamiana* leaves. Yellow and red fluorescence was observed with Confocal Fluorescence Microscope as described in the subcellular localization assays.

## Results

### Nucleotide and Protein Sequence Characteristics of Lotus WRKY70s

Previously, two independent studies have identified NnWRKY70a (Nnu_24385) and NnWRKY70b (Nnu_12194) as key candidate regulators of the lotus BIA biosynthesis based on a positive correlation between their expression and BIA accumulation in lotus (Deng et al., 2018; Meelaph et al., 2018). To evaluate the possible roles of these two WRKYs in regulating the lotus BIA biosynthesis, we first analyzed their nucleotide and amino acid sequence characteristics. *NnWRKY70a* was found to be located on the lotus chromosome 5, which contains a 981 nucleotide (nt) open reading frame (ORF), encoding a protein with a calculated molecular weight of 36.5 kDa. *NnWRKY70b* was located on chromosome 2, which has a 990 nt ORF, encoding a 37.06 kDa protein. Pairwise sequence alignment showed that the two NnWRKY70s shared high-sequence identity, with nucleotide and amino acid sequence similarity of 70.6 and 74.4%, respectively.

**Figure 2:**
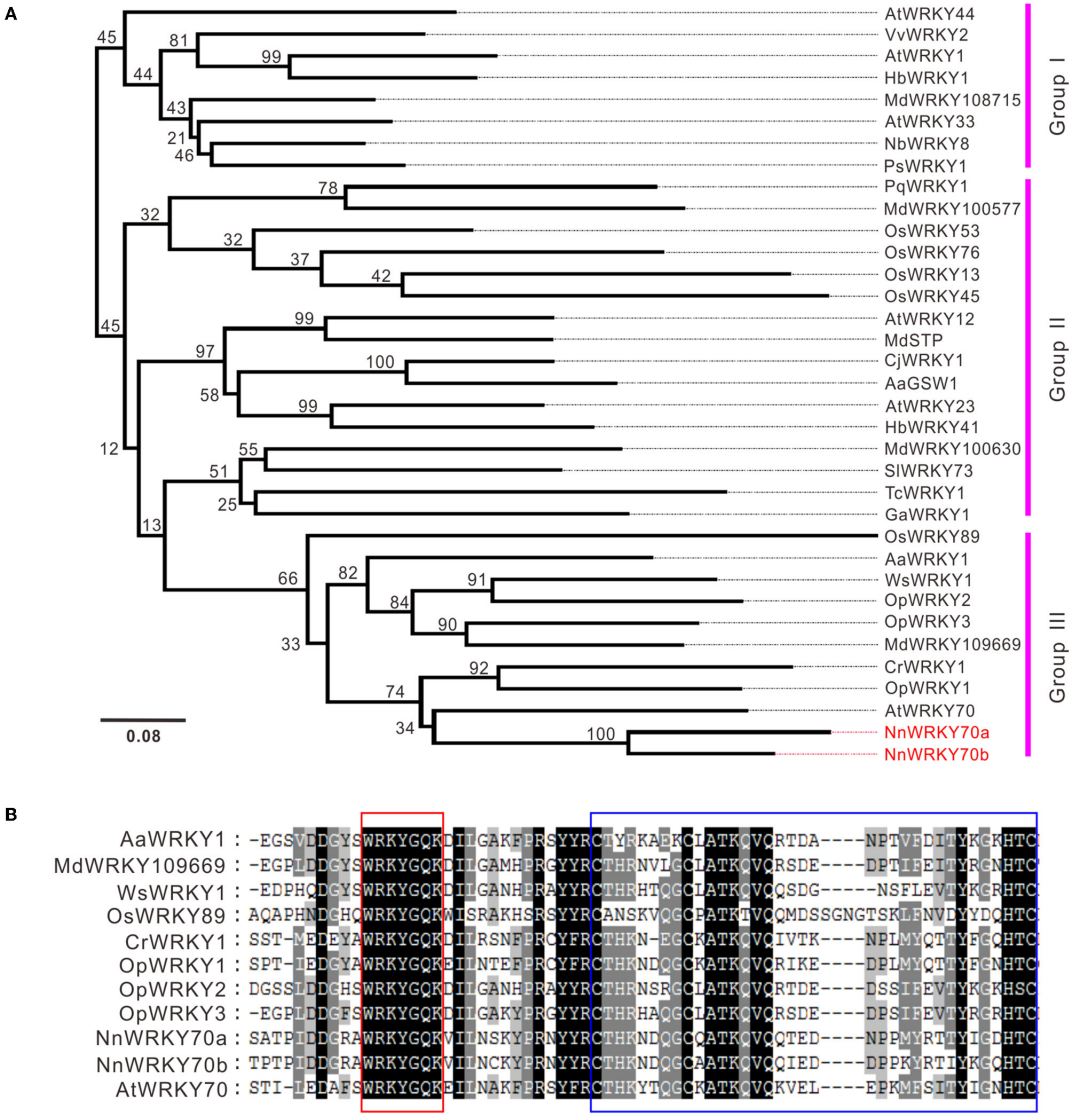
Nucleotide and amino acid sequence characteristics of lotus WRKY70s. **(A)** Phylogenetic analysis of NnWRKY70a and NnWRKY70b with other WRKYs involved in the biosynthesis of plant secondary metabolites. Protein names, species, and GenBank accession numbers are listed in Supplementary Table S2. **(B)** Protein sequence alignment of the selected group III WRKY members. Conserved WRKY and zinc-finger domains are highlighted in red and blue boxes, respectively.

A phylogenetic analysis was conducted using two NnWRKY70s and other previously characterized WRKYs involved in the regulation of plant secondary metabolism (Supplementary Table S2). Two NnWRKYs clustered together with group III members (Figure 2A). NnWRKY70s showed the highest sequence similarity with the AtWRKY70, followed by CrWRKY1 and OpWRKY1. Both CrWRKY1 and OpWRKY1 are group III WRKYs involved in regulating MIA biosynthesis in *Catharanthus roseus* and *Ophiorrhiza pumila*, respectively (Suttipanta et al., 2011; Xu et al., 2020). Similar to the other group III WRKYs, both NnWRKY70s contained a core WRKYGQK heptapeptide and a conserved CX_7_CX_23_HXC zinc-finger motif in their WRKY domains (Figure 2B). Therefore, NnWRKY70a and NnWRKY70b were both typical group III WRKY proteins similar to AtWRKY70.

### Expression Profiling and Subcellular Localization of NnWRKY70s

**Figure 3:**
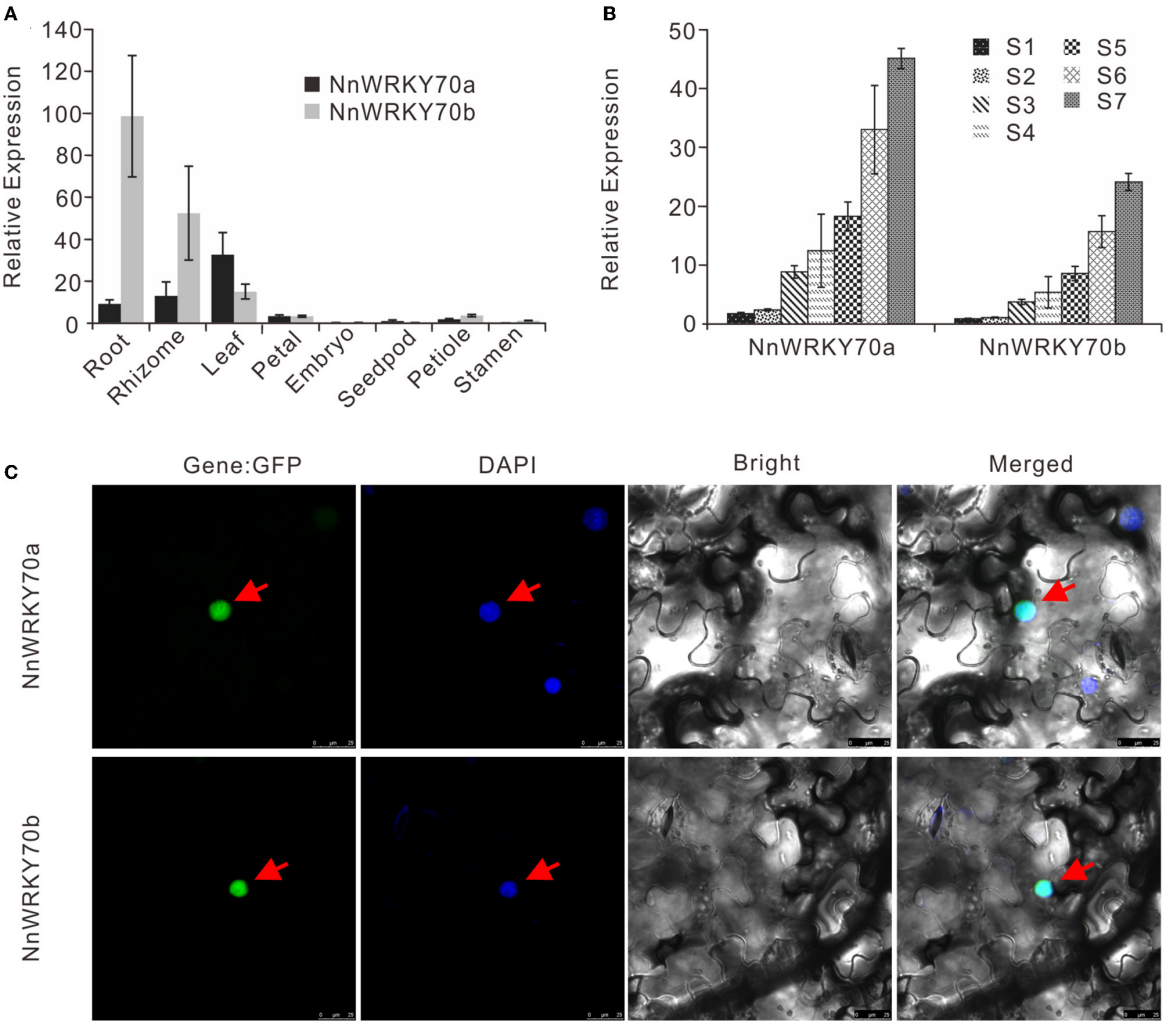
Expression profiling and subcellular localization of NnWRKY70s. **(A)** Spatial expression profiling of *NnWRKY70*s in different lotus organs. **(B)** Temporal expression profiling of *NnWRKY70*s in seven developmental stages of lotus leaf (S1–S7). Data of **(A,B)** are mean ± SE (*n* = 3). **(C)** Subcellular localization of NnWRKY70a and NnWRKY70b in *N. benthamiana* leaves. Panels from left to right refer to GFP, DAPI, bright, and merged images, respectively. Protein fusions co-localized with DAPI markers are marked with red arrows.

Next, we analyzed the spatial expression patterns of *NnWRKY70* genes in the lotus variety “Qiuxing” by quantitative real-time PCR. The two *WRKY70*s showed quite distinct expression patterns (Figure 3A). Overall, *NnWRKY70b* was predominantly expressed in the lotus root, followed by the rhizome and leaf. In contrast, *NnWRKY70a* had the highest transcript levels in the lotus leaf, where mono-BIA are primarily accumulated.

Their expression profiling was also evaluated in seven lotus leaf developmental stages (Figure 3B). Two lotus *NnWRKY70* genes showed quite similar expression patterns, with both consistently increasing throughout the tested developmental stages. This expression pattern followed well with our previously reported BIA accumulation pattern in lotus leaves (Deng et al., 2016). In addition, we carried out a subcellular localization assay by fusing the coding regions of *NnWRKY70s* with a GFP reporter (Figure 3C). The GFP fluorescence of both NnWRKY70a:GFP and NnWRKY70b:GFP fused proteins co-localized with the DAPI-stained nucleus, demonstrating an obvious nuclear localization of both NnWRKY70a and NnWRKY70b proteins.

### NnWRKY70s Are JA-Responsive TFs That Transactivate BIA Pathway Gene Promoters

**Figure 4:**
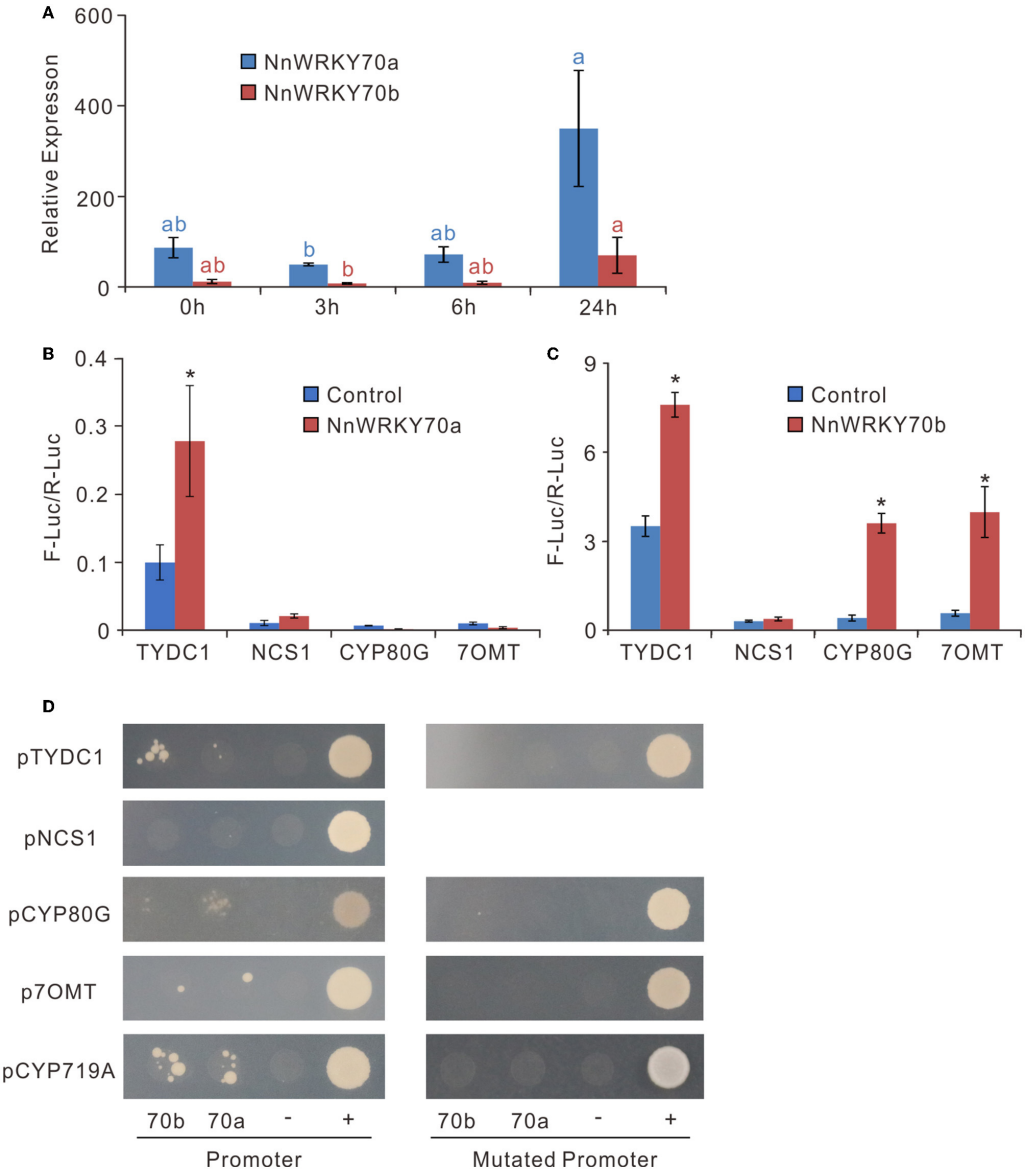
NnWRKY70s are JA responsive and can activate the transcription of BIA biosynthetic genes. **(A)** Expression of *NnWRKY70a* and *NnWRKY70b* in lotus leaves in response to MeJA treatment. Lotus leaves were treated with MeJA at the developmental stage 4. **(B,C)** Dual-luciferase assays assessing the effect of NnWRKY70a and NnWRKY70b on activating the BIA pathway gene promoters. **(D)** Yeast one-hybrid assays checking the interactions between NnWRKY70a/NnWRKY70b transcription factors and BIA pathway gene promoters. Y1HGold bait strains transformed with pGADT7 prey vectors were cultured on SD/-Leu medium supplied with 100 ng/mL AbA. “70b”, NnWRKY70b; “70a”, NnWRKY70a; “-”, negative control transformed with empty AD vector; “+”, positive control (p53-AbAi+AD-p53); Mutated promoters, BIA pathway gene promoters with W-boxes removed. Data of **(A**–**C)** are mean ± SE (*n* = 3). Lower case letters and asterisks (*) represent significant differences at *P* < 0.05.

JA is known to trigger most biosynthetic pathways leading to secondary metabolites by activating the JA-responsive TFs (Wasternack and Strnad, 2019; Yamada et al., 2020). To verify whether *NnWRKY70*s are JA responsive, we checked their expression in the MeJA-treated samples, wherein the lotus leaves were treated with 100 μM MeJA at the developmental stage 4 (Li et al., 2019). MeJA significantly induced both *NnWRKY70* genes, with their expression increasing approximately 5-fold at 24 h after treatment (Figure 4A). This was consistent with the increased BIA content in the lotus leaves, a 15–30% increase between 3 and 6 h, and an approximately 50% increase at 24 h after the MeJA treatment (Li et al., 2019).

Next, we screened the promoter regions of the BIA biosynthetic genes, including *NnTYDC1, NnNCS1, NnCYP80G*, and *Nn7OMT*, for potential binding sites of WRKY TFs. Conserved W-box (TTGACT) was found in the 2 kb promoter regions of *NnTYDC1, NnCYP80G*, and *Nn7OMT*, but not of the *NnNCS1* gene. To further confirm whether NnWRKY70a and NnWRKY70b could activate the three BIA gene promoters, a dual-luciferase assay was conducted. Transient overexpression of NnWRKY70a significantly enhanced the pNnTYDC1-driven firefly luciferase transcription but had no effect on the other three promoters (Figure 4B). In contrast, transient overexpression of NnWRKY70b was able to activate three promoters, including *pNnTYDC1, pNnCYP80G*, and *pNn7OMT2* (Figure 4C).

To further test whether NnWRKY70a and NnWRKY70b bind directly to these promoters, we conducted the yeast one-hybrid assays for putative BIA structural gene promoters and their corresponding mutants (with W-box removed). NnWRKY70b was bound to *pNnTYDC1* and *pNnCYP719A* and weakly bound to *pNnCYP80G* and *pNn7OMT* (Figure 4D). In contrast, NnWRKY70a was bound obviously to the *NnCYP80G* and *NnCYP719A* promoters, while weakly bound to the *NnTYDC1* and *Nn7OMT* promoters. However, neither NnWRKY70b nor NnWRKY70a bound to the *NnNCS1* promoter. Markedly, when the W-boxes were removed from these promoters, none of them were bound by NnWRKY70a or NnWRKY70b, indicating a specific binding of NnWRKY70a or NnWRKY70b to the W-box *cis*-elements in the BIA biosynthetic gene promoters. Taken together, these results suggest that both NnWRKY70a and NnWRKY70b can bind and activate the BIA pathway gene promoters.

### Overexpression of *NnWRKY70s* Enhances BIA Accumulation in Lotus

**Figure 5:**
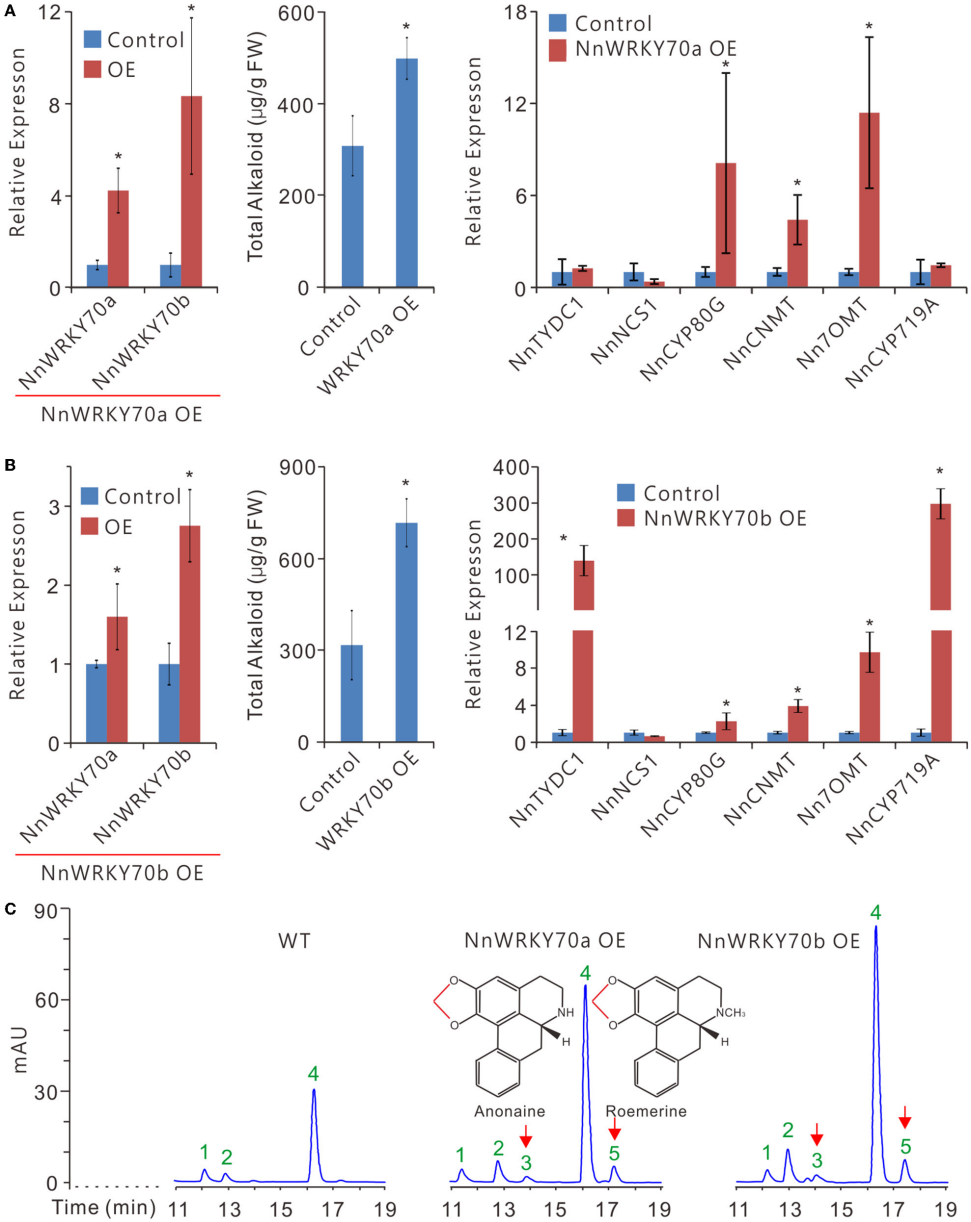
Transient overexpression of *NnWRKY70a* and *NnWRKY70b* significantly enhances BIA accumulation in lotus petals. **(A,B)** Effects of NnWRKY70 overexpression on BIA biosynthesis in lotus. Left panel, expression of *NnWRKY70a* and *NnWRKY70b*; Middle panel, BIA concentration; Right panel, expression of BIA pathway genes in lotus petals overexpressing *NnWRKY70*s. **(C)** HPLC scanning of BIA constituents and their abundance in lotus petals. Data are means ± SE (*n* = 3). Asterisks (*) represents significant differences at *P* < 0.05. Peak signals 1-5 represent *N*-nornuciferine, *O*-nornuciferine, anonaine, nuciferine, and roemerine alkaloids, respectively. Chemical bonds marked in red are methylenedioxy bridges formed by the catalytic activity of NnCYP719A.

To determine *in planta* whether NnWRKY70a and NnWRKY70b activate the BIA biosynthesis in lotus, an *Agrobacterium*-mediated transient overexpression assay was conducted. Given that the lotus leaves were almost unable to be infiltrated, we performed agroinfiltration in the lotus petals in the “Qiuxing” variety, which is known to accumulate moderate levels of BIAs (Deng et al., 2016, 2022). As a result, a significant 4.2-fold increase in the transcript levels of *NnWRKY70a* was observed in the petals infiltrated with *Agrobacterium* carrying an overexpression vector (Figure 5A). Similarly, an approximately 2-fold increase in the expression of *NnWRKY70b* was observed in the *NnWRKY70b* overexpressing petals (Figure 5B). Interestingly, the overexpression of either *NnWRKY70a* or *NnWRKY70b* significantly promoted the transcription of the other members (Figures 5A,B), indicating a possible inter-activation ability between the two NnWRKY70s.

Moreover, the overexpression of the two *NnWRKY70*s enhanced the BIA contents in the lotus petals (Figures 5A,B). Petals overexpressing *NnWRKY70a* had approximately 60% increase in the total BIA content, whereas those overexpressing *NnWRKY70b* exhibited an even higher increase of about 126%. Consistently, increased BIA contents in petals overexpressing *NnWRKY70*s were accompanied by significant elevation of BIA pathway genes. Of the six tested BIA biosynthetic genes, three, including *NnCYP80G, NnCNMT*, and *Nn7OMT*, were markedly enhanced in the petals overexpressing *NnWRKY70a*, with approximately 8.1-, 4.4-, and 11.4-fold increase in the expression, respectively (Figure 5A). In contrast, a more dramatic increase in the gene expression was observed in the petals overexpressing *NnWRKY70b*, with *NnTYDC1, NnCYP80G, NnCNMT, Nn7OMT*, and *NnCYP719A* genes exhibiting approximately 151.4-, 2.2-, 3.9-, 9.7-, and 309.3-fold increase in their expression, respectively (Figure 5B). This further revealed that *NnWRKY70b* overexpression could induce a more dramatic increase in the BIA accumulation.

It was known that lotus petals mainly accumulate five types of BIAs, nuciferine, *N*-nornuciferine, *O*-nornuciferine, roemerine, and anonaine, with nuciferine as a predominant one (Deng et al., 2016). Of the six tested BIA pathway enzymes, NnTYDC1, NnNCS1, NnCYP80G, and NnCNMT are located upstream and are involved in the biosynthesis of all the five BIAs in lotus petals (Figure 1). In contrast, Nn7OMT is especially involved in the production of nuciferine, *N*-, and *O*-nornuciferine, whereas NnCYP719A mainly catalyzes the production of roemerine and anonaine. An HPLC analysis detected three BIA peaks corresponding to *N*-nornuciferine (peak 1), *O*-nornuciferine (peak 2), and nuciferine (peak 4) in the uninfiltrated “Qiuxing” petals (Figure 5C). The overexpression of *NnWRKY70*s significantly increased the accumulation of all the three detected BIAs (Supplementary Figure S1). Interestingly, the two additional peaks, representing anonaine (peak 3) and roemerine (peak 5), respectively, were evidently detected (Figure 5C; Supplementary Figures S1A,B). This is obviously due to the significant elevation of *NnCYP719A* expression in the lotus petals. Taken together, our results demonstrate that both NnWRKY70a and NnWRKY70b are involved in the BIA biosynthesis *in planta*.

### NnWRKY70b Physically Interacts With NnWRKY70a, NnWRKY53b, and NnJAZ1 Proteins

**Figure 6:**
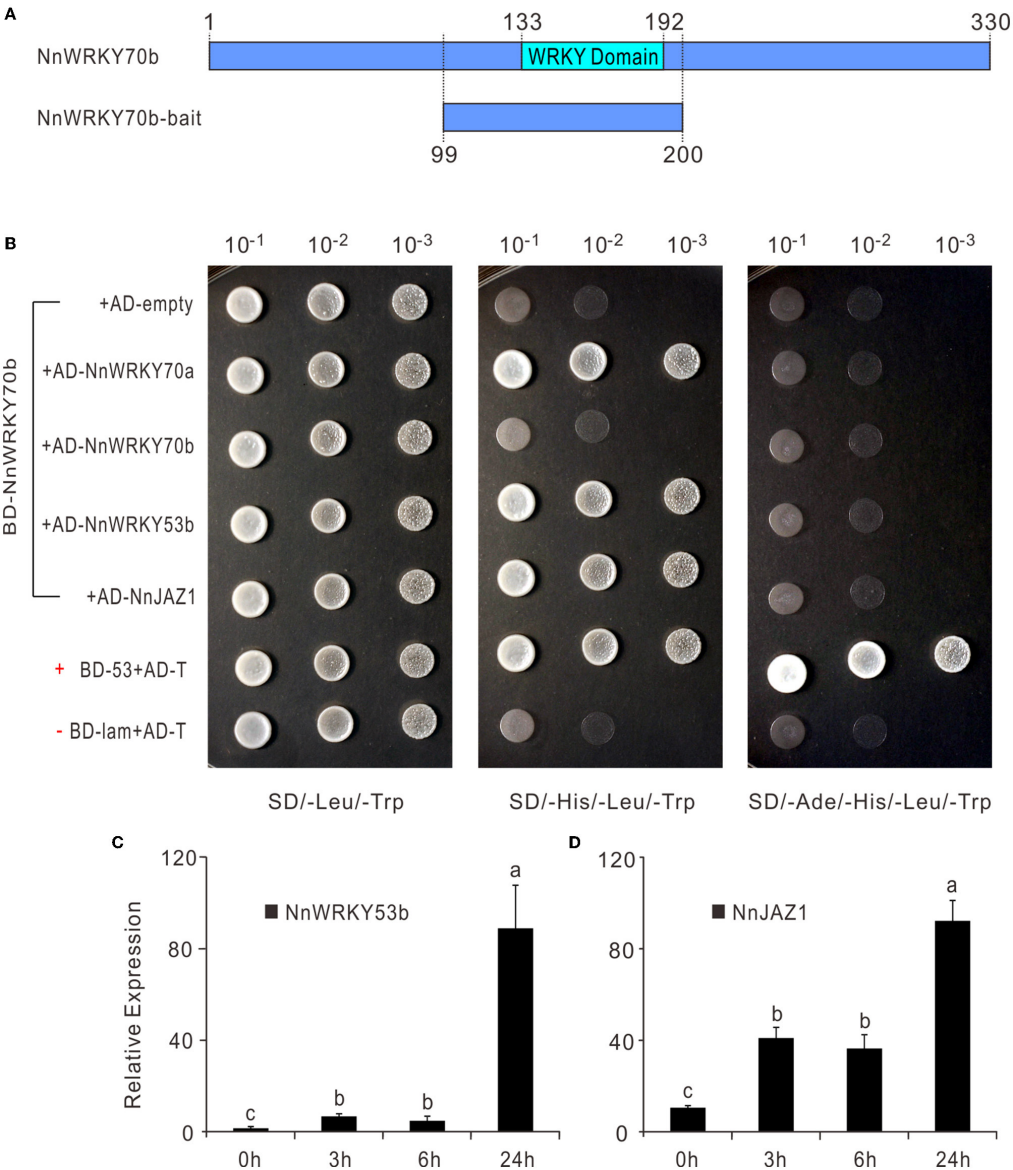
NnWRKY70b regulates the lotus BIA biosynthesis by interacting with NnWRKY70a, NnWRKY53b, and NnJAZ1 proteins. **(A)** Schematic showing the truncated NnWRKY70b bait used in the yeast two-hybrid assay. **(B)** Yeast two-hybrid assay showing the interaction between NnWRKY70b and other proteins. AD and BD represent the two yeast vectors of pGADT7 and pGBKT7, respectively. Control vectors BD-53 and BD-lam carry the murine p53 and the lamin respectively, while AD-T carries SV40 larger T-antigen. p53 and T-antigen are known to interact in the yeast two-hybrid assay. Thus, a combination of BD-53+AD-T was used as a positive control, while the un-interactional BD-Lam+ AD-T was set as a negative control. **(C,D)** Expression of *NnWRKY53b* and *NnJAZ1* in response to MeJA treatment. MeJA treatment was conducted in lotus leaves at the developmental stage 4. Data are means ± SE (*n* = 3). Lower case letters represent significant differences at *P* < 0.05.

As shown above, NnWRKY70a and NnWRKY70b might interregulate the expression of each other. Previous studies also showed that the group II and III WRKY members in *Arabidopsis* often function by forming homodimers or heterodimers with other WRKY TFs (Xu et al., 2006; Besseau et al., 2012). To investigate the possible interaction between NnWRKY70s and other proteins in regulating the lotus BIA biosynthesis, we performed a yeast two-hybrid cDNA library screen assay. NnWRKY70b was selected as a bait due to its significant ability to regulate the BIA biosynthesis. The bait construct harboring full-length NnWRKY70b displayed a strong autoactivation activity. Thus, truncated NnWRKY70b bait was used to screen the library. In this bait, the WRKY and zinc-finger domains were kept, while its activation domain was dropped out (Figure 6A). As a result, a total of 32 proteins potentially interacting with NnWRKY70b were identified (Supplementary Table S3). Of these interactions, NnWRKY70b vs. NnWRKY70a, NnWRKY70b vs. NnWRKY53b, and NnWRKY70b vs. NnJAZ1 were proved to be genuine during the subsequent yeast two-hybrid assays.

**Figure 7:**
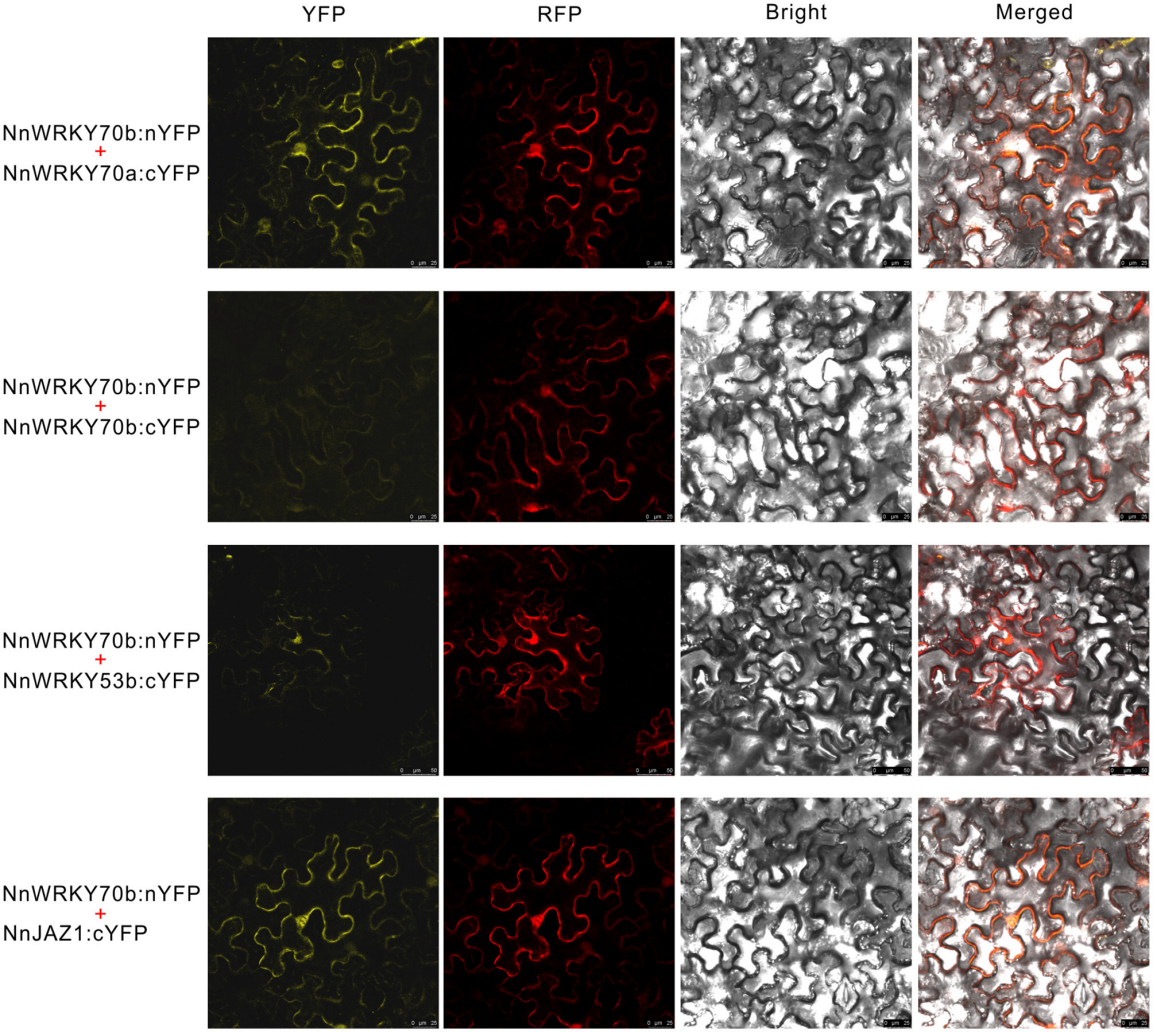
Bimolecular Fluorescence Complementation (BiFC) assays showed that NnWRKY70b could interact with NnWRKY70a, NnWRKY53b, and NnJAZ1 *in vivo*. BiFC assays were conducted with a 2in1 system based on the splitting of enhanced yellow fluorescent protein (EYFP). Two fusion proteins were expressed with the same pBiFCt-2in1-CN vector, and a soluble monomeric red fluorescent protein (mRFP) was expressed in the same vector as an internal marker for transformation and expression control.

The truncated NnWRKY70b bait could not interact with NnWRKY70b itself but positively interacted with NnWRKY70a, and another group III type protein NnWRKY53b (Figure 6B). NnWRKY70b also interacted with NnJAZ1, a lotus JAZ repressor protein. Both NnWRKY53b and NnJAZ1 were also JA responsive and displayed similar expression patterns as NnWRKY70a and NnWRKY70b under the JA treatment (Figures 6C,D). These interactions were also confirmed *in vivo* by the BiFC assays. The EYFP fluorescence was detected in the living *N. benthamiana* epidermal cells infiltrated with pBiFCt-2in1 vectors carrying coding sequence of *NnWRKY70b* and *NnWRKY70a, NnWRKY70b* and *NnWRKY53b*, and *NnWRKY70b* and *NnJAZ1* (Figure 7).

Next, we evaluated whether NnWRKY53b interacts with NnJAZ1 and the capacity of NnWRKY53b to activate BIA pathway gene promoters. The yeast two-hybrid assay showed that NnWRKY53b did not physically interact with NnJAZ1 (Supplementary Figure S2). A transient trans-activation assay in tobacco indicated that NnWRKY53b alone can activate *pNnTYDC1* (Supplementary Figure S3). A combination of NnWRKY70b and NnWRKY53b, however, did not show enhanced activation abilities on BIA gene promoters, in compared to either of the singletons. These results suggest NnWRKY70b may regulate the lotus BIA biosynthesis through the JA-signaling pathway. The interaction between NnWRKY70b and NnWRKY53b, however, did not enhance the BIA biosynthesis.

## Discussion

Lotus is a large aquatic plant that has long been used as a traditional herb (Mukherjee et al., 2009). BIAs, including nuciferine and liensinine, are the major bioactive components in lotus tissues and have been proved to have significant pharmacological properties, such as antioxidant, anti-cancer, anti-HIV, anti-inflammatory, anti-obesity, and hepatoprotective effects (Sharma et al., 2017). To date, the studies on the lotus BIAs have been mainly focused on their structural elucidation, component isolation, and pharmacological function characterization, but little is still known about the regulation of their biosynthesis, with only a few available reports on the BIA biosynthesis (Vimolmangkang et al., 2016; Deng et al., 2018; Menendez-Perdomo and Facchini, 2020). This study presents a comprehensive functional analysis of the two JA-responsive WRKY TFs, NnWRKY70a and NnWRKY70b, and demonstrates their roles in the positive regulation of the BIA biosynthesis in lotus through transcriptional activation of BIA structural genes.

A phylogenetic analysis revealed that NnWRKY70a and NnWRKY70b are typical group III WRKYs, clustered closely with AtWRKY70 and harboring a conserved WRKY DNA binding domain, with a WRKYGQK heptapeptide in the N-terminal and a C2HC type zinc-finger motif in the C-terminal (Figure 2). Two NnWRKY70s shared over 70% amino acid sequence identity and were highly syntenic, suggesting that they probably evolved from a common ancestor during a recent whole genome duplication event in lotus (Ming et al., 2013; Gui et al., 2018; Li et al., 2019).

Previous studies have demonstrated a strong correlation in their expression and the accumulation of BIA in lotus leaves (Deng et al., 2018; Meelaph et al., 2018). Here, we further showed that exogenous application of MeJA in the lotus leaves significantly induced their expression and the accumulation of BIA in the lotus leaves. The dual-luciferase assays showed that NnWRKY70a could activate the *NnTYDC2* promoter, while NnWRKY70b activated three BIA pathway gene promoters (Figure 4). Furthermore, the overexpression of the two WRKYs in lotus petals significantly elevated the BIA biosynthetic gene expression and the BIA accumulation. The yeast one-hybrid assays showed that NnWRKY70a and NnWRKY70b can specifically bind to the W-box *cis*-elements of BIA pathway gene promoters. These observations suggest that both NnWRKY70a and NnWRKY70b transcriptionally activate the BIA structural genes and positively regulate the lotus BIA biosynthesis.

Notably, the two NnWRKY70 TFs are unevenly involved in the regulation of BIA biosynthesis in the lotus. A stronger activation of BIA biosynthesis was observed for NnWRKY70b than NnWRKY70a. For example, NnWRKY70b significantly activated the promoters of the three structural genes, including *NnTYDC, NnCYP80G*, and *Nn7OMT*, whereas NnWRKY70a could only activate the promoter of *NnTYDC*. In addition, more BIA structural genes were upregulated in the lotus petals overexpressing NnWRKY70b than in those overexpressing NnWRKY70a (Figure 5). In addition to the three genes (*NnCYP80G, NnCNMT*, and *Nn7OMT*) activated by the NnWRKY70a overexpression, the NnWRKY70b overexpression also strongly boosted the expression of *NnTYDC1* and *NnCYP719a*, with their expression increased over 100 folds. Moreover, approximately 120% increase in the accumulation of BIA was observed in the petals overexpressing NnWRKY70b, while about 60% increase was observed for the petals overexpressing NnWRKY70a. This is similar to the previous observations: two closely related group III WRKY proteins in *O. Pumila*, OpWRKY2 and OpWRKY3, showed varied regulatory effects on the biosynthesis of an anticancer drug camptothecin (Wang et al., 2019; Hao et al., 2021). It has been reported that WRKY70 in *Arabidopsis* acted as a convergent node for salicylic acid (SA) and JA-mediated defense signal pathways, and was intensively involved both in biotic- and abiotic-stress responses (Li et al., 2004, 2013, 2017). Thus, besides their role in regulating the BIA biosynthesis, NnWRKY70s in lotus could also potentially be associated with lotus's innate immunity.

Interestingly, *NnNCS1*, a crucial BIA biosynthetic gene in the lotus (Vimolmangkang et al., 2016), was not activated by either of the two NnWRKY70 TFs. This could be attributed to the lack of a W-box *cis*-element binding site in its promoter (Eulgem et al., 2000). W-box was identified within 2 kb promoter regions of the four BIA structural genes, including *NnTYPC, NnCYP80G, Nn7OMT*, and *NnCYP719A*, but not in the promoters o*f NnNCS1* and *NnCNMT*. The yeast one-hybrid assays showed that NnWRKY70s can bind to the four tested BIA gene promoters that contain W-boxes (Figure 4). However, *NnCNMT*, despite the lacking of W-box in its promoter, was significantly induced by the overexpression of both NnWRKY70s. Thus, the two NnWRKY70 TFs may have binding preferences other than W-box; otherwise, they may have activated other TFs that can positively regulate *NnCNMT*. Previous studies have shown that some WRKY TFs had indeed altered the binding preference of WK (TTTCCAC) and WT (GGACTTTC) boxes (Machens et al., 2014; Kanofsky et al., 2017). Further studies are still needed to explore the exact binding site of NnWRKY70s. Intriguingly, NnWRKY70b overexpression significantly also enhanced the expression of *NnCYP719A*. As a result, the accumulation of roemerine and anonaine alkaloids was significantly elevated in lotus petals (Figure 5). This further verified the role of NnCYP719A in catalyzing the formation of methylenedioxy bridges in roemerine and anonaine biosynthesis (Ikezawa et al., 2003).

Both the yeast two-hybrid and BiFC assays further revealed possible interactions between NnWRKY70b and other proteins (Figures 6, 7). NnWRKY70b did not physically interact with itself, but interacted with two group III WRKY TFs, NnWRKY70a and NnWRKY53b, suggesting that NnWRKY70b may activate BIA pathway genes through forming protein complexes. Notably, although NnWRKY53b activated two BIA pathway gene promoters, it did not physically interact with NnJAZ1. The combination of NnWRKY70a and NnWRKY53b did not show gained effects on the activation of BIA structural gene promoters. AtWRKY70 and AtWRKY53 are known to negatively regulate the leaf senescence and drought tolerance in *Arabidopsis* (Li et al., 2004; Miao and Zentgraf, 2007; Sun and Yu, 2015). In contrast to the JA-inducible characteristic of NnWRKY70s and NnWRKY53b, the homologous *Arabidopsis* genes were reported to be suppressed by the MeJA treatment. Thus, functions of WRKY70 and WRKY53 may have diverged in the two species.

In addition, NnWRKY70b also interacted with NnJAZ1. JAZ family proteins are the key regulators in the JA signaling pathway, which interact with and repress TFs that regulate plant secondary metabolism (Pauwels and Goossens, 2011; Nagels Durand et al., 2016). Bioactive JAs can be sensed by the F-box protein Coronatine Insensive1 (COI1), the recognition component of the E3 ubiquitin ligase complex SCF^COI1^. As a result, JAZ proteins are ubiquitinated and degraded *via* the ubiquitin-26S proteasome pathway (Thines et al., 2007). Given the positive interaction between NnWRKY70b and NnJAZ1, it is reasonable to speculate that the JA-responsive NnWRKY70b also regulates the lotus BIA biosynthesis via the JA-signaling pathway. It is however should be noticed that the EYFP fluorescence raised from the interacted protein complex was not located in the nucleus, where the interaction was supposed to happen. This is probably due to the slow maturation property of EYFP and the irreversible BiFC complex, which resulted in the discrepancy between the bimolecular fluorescent and the site of protein interactions, as has been reviewed and pointed out previously (Miller et al., 2015).

**Figure 8:**
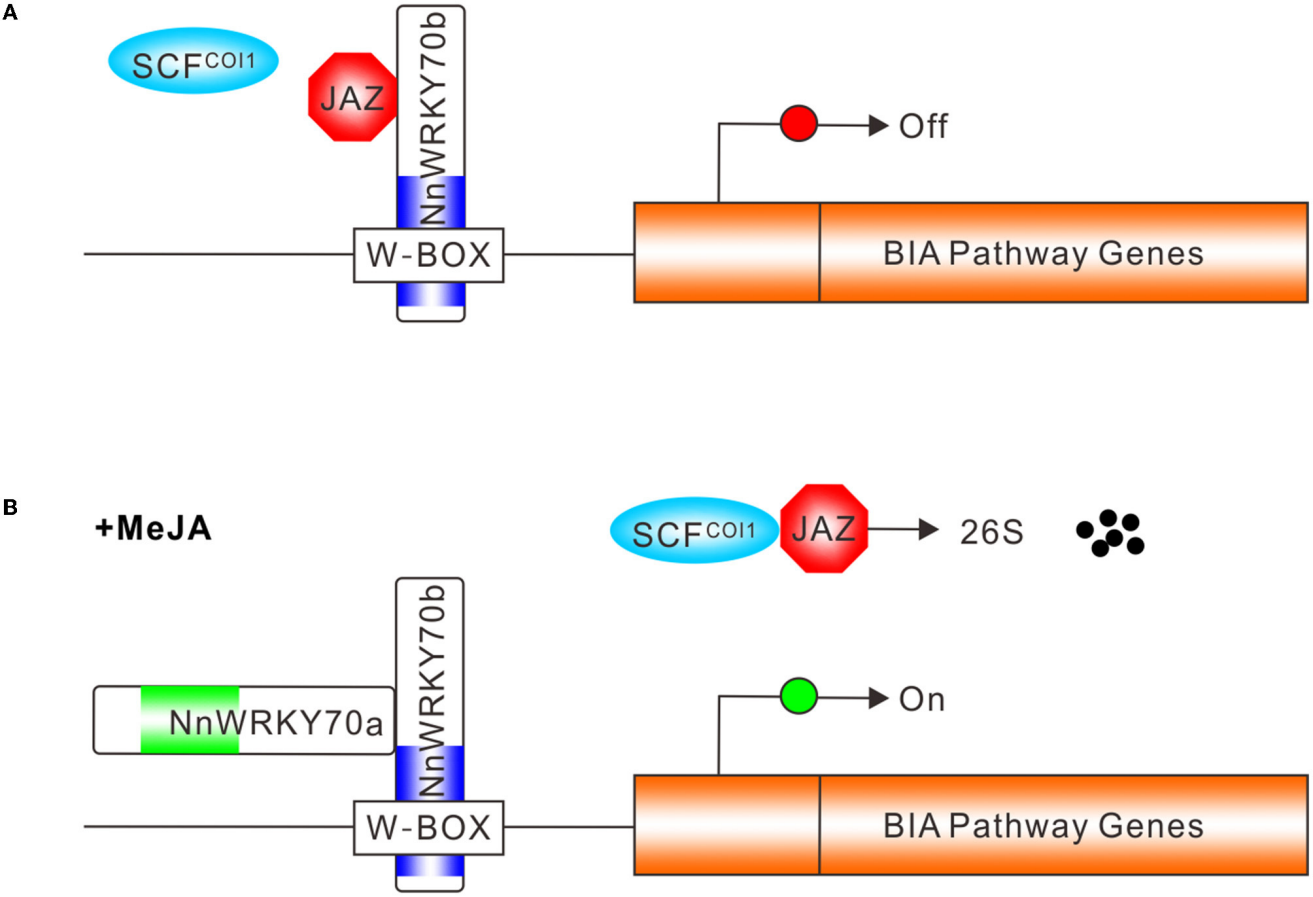
Simplified model of NnWRKY70b TF action in regulating lotus BIA biosynthesis. **(A)** In the absence of JA, JAZ proteins bind to NnWRKY70b and repress its activity, resulting in the inactivation of BIA pathway genes in the lotus. **(B)** In the presence of JA, the SCF^COI1^ complex facilitates the ubiquitin-26S mediated JAZ degradation. The NnWRKY70b protein is then released and forms protein complexes with another group III WRKY proteins to positively regulate the BIA pathway genes, through binding the W-box *cis*-element in their promoters. Blue and green boxes represent WRKY DNA binding domains in NnWRKY70a and NnWRKY70b, respectively.

Based on the above findings, we propose a summary model depicting the possible action mode of NnWRKY70 TFs in regulating the lotus BIA biosynthesis. In the absence of JAs, JAZ proteins bind to NnWRKY70b and suppress its activity (Figure 8A), whereas, in the presence of bioactive JAs, JAZ proteins are degraded *via* the SCF^COI1^-mediated ubiquitin-26S proteasome pathway (Figure 8B). The unbound NnWRKY70b then forms protein complexes with other co-factors such as NnWRKY70a, and transactivate BIA biosynthetic genes by binding to the W-box *cis*-elements in their promoters. Transcription activators can usually activate the expression of a range of pathway genes, and massively increase the secondary metabolite production. Our present findings demonstrate the positive regulatory role of NnWRKY70 TFs in activating the biosynthesis of BIA in the lotus and providing a feasible strategy for improving the BIA production through TF-based genetic engineering.

## Data Availability Statement

The original contributions presented in the study are included in the article/Supplementary Material, further inquiries can be directed to the corresponding author/s.

## Author Contributions

JL and XD conceived and designed the experiments. JL, YLi, XD, MD, SL, SC, and MZ performed the experiments. JL wrote the paper. XD, MY, DY, YLiu, and DT revised the manuscript. All authors contributed to the article and approved the submitted version.

## Funding

This project was supported by funds received from the National Natural Science Foundation of China (Grant Nos. 31700262 and 32070336), the Fundamental Research Funds for the Central Universities (WUT: 2020IB031), the National Innovation and Entrepreneurship Training Program for College Students (Grant No. S202010497060), the Open Fund of Shanghai Key Laboratory of Plant Functional Genomics and Resources, and the Natural Science Foundation of Shandong Province (No. ZR2021MC163).

## Conflict of Interest

The authors declare that the research was conducted in the absence of any commercial or financial relationships that could be construed as a potential conflict of interest.

## Publisher's Note

All claims expressed in this article are solely those of the authors and do not necessarily represent those of their affiliated organizations, or those of the publisher, the editors and the reviewers. Any product that may be evaluated in this article, or claim that may be made by its manufacturer, is not guaranteed or endorsed by the publisher.
