# Supplementary material for: Jasmonate-Responsive Transcription Factors NnWRKY70a and NnWRKY70b Positively Regulate Benzylisoquinoline Alkaloid Biosynthesis in Lotus (Nelumbo nucifera)
Source: Front Plant Sci. 2022 Jun 15;13:862915. doi: 10.3389/fpls.2022.862915 (PMC9240598; doi:10.3389/fpls.2022.862915)
Supplement: Supplementary file 1 [file Data_Sheet_1.docx]

**Table S1** Primers used for quantitative RT-PCR.

| **No** | **Name** | **Sequence (5’-3’)** |
| --- | --- | --- |
| 1 | qPCR_TYDC1_F | GCAAGACGTGCAGAAAGAAATTATCC |
| 2 | qPCR_TYDC1_R | GCAGCAGGCGAAGACATCCAA |
| 3 | qPCR_NCS1_F | GAGCTGCCCAGACTCTTCGTC |
| 4 | qPCR_NCS1_R | TACCTTCTCCCGGTGTTGATG |
| 5 | qPCR_CYP80G_F | AGCGTCCGAATTAAGGGCTAT |
| 6 | qPCR_CYP80G_R | ATCAGTTCCGATACTTTCTCCTCT |
| 7 | qPCR_CNMT_F | CCATCAAGACCGACTTACCAA |
| 8 | qPCR_CNMT_R | TCACAGTACAGCTCCAGCATAG |
| 9 | qPCR_CYP719A_F | ACAAGCGGCTGTGAAGGAGAC |
| 10 | qPCR_CYP719A_ | CATTTGGATTATAGTGTATAGCGTGAAG |
| 11 | qPCR_7OMT_F | ATGAAGGAATGGCTTGTGATAC |
| 12 | qPCR_7OMT_R | TCGGCAATGACATGAGGTAGA |
| 13 | qPCR_ACTIN_F | CTCCGTGTTGCCCCTGAAG |
| 14 | qPCR_ACTIN_R | CCAGCAAGGTCCAACCGAAG |
| 15 | qPCR_WRKY70a_F | CAGGAATGCAAAGAGGTGGAGA |
| 16 | qPCR_WRKY70a_R | ACCTGAGTACACGCCCGAGAT |
| 17 | qPCR_WRKY70b_F | CAGGAATGCAAAGAGGTGGAGA |
| 18 | qPCR_WRKY70b_R | ACCTGAGTACACGCCCGAGAT |
| 19 | WRKY70bY_F | CCTTAATTAAATGGACACAACTGTTGAATCTC |
| 20 | WRKY70bY_R | TTGGCGCGCCAAACCGGAAAAAAGTCATCATC |
| 21 | WRKY70aY_F | CCTTAATTAAATGGGGACAGTGGAATCTCCGT |
| 22 | WRKY70aY_R | TTGGCGCGCCACATTGAAACCTGAAAGATGTCC |
| 23 | Luc_WRKY70a_IF | CGGAATTCATGGGGACAGTGGAATCTCCGT |
| 24 | Luc_WRKY70a_IR | GCTCTAGATTACATTGAAACCTGAAAGATGTCC |
| 25 | Luc_WRKY70b_IF | CGGAATTCATGGACACAACTGTTGAATCTC |
| 26 | Luc_WRKY70b_IR | GCTCTAGATTAAACCGGAAAAAAGTCATCATC |
| 27 | Luc_TYDC1_pF | CGCGGATCCCCCAAGTTGTAGACCCGTTAG |
| 28 | Luc_TYDC1_pR | CATGCCATGGGCTGGAGATGGAGATGGAGATG |
| 29 | Luc_NCS1_pF | CGCGGATCCACACGCTATCACAAACCTACCT |
| 30 | Luc_NCS1_pR | CATGCCATGGCTTTTTCTTCGTTGGAAGGATGCA |
| 31 | Luc_CYP80G_pF | CGCGGATCCCCTTACTTAGTATGGGGTCATTG |
| 32 | Luc_CYP80G_pR | CATGCCATGGTAGAGTCTATAGAGAAGATGTAGTAC |
| 33 | Luc_7OMT2_pF | CGCGGATCCTCGGACTGGAAACTGAGAACC |
| 34 | Luc_7OMT2_pR | CATGCCATGGGGTTGTTCTCTTTTTTAGGACTGC |
| 35 | AD-NnWRKY70a-F | CAGATTACGCTCATATGATGGGGACAGTGGAATCTCC |
| 36 | AD-NnWRKY70a-R | TGCTTGGGTGGAATTCTTACATTGAAACCTGAAAGATGTC |
| 37 | AD-NnWRKY70b-F | CAGATTACGCTCATATGATGGACACAACTGTTGAATCTCC |
| 38 | AD-NnWRKY70b-R | TGCTTGGGTGGAATTCTTAAACCGGAAAAAAGTCATCATC |
| 39 | AD-NnWRKY53b-F | CAGATTACGCTCATATGATGGAGAACGCCGGGGAATG |
| 40 | AD-NnWRKY53b-R | TGCTTGGGTGGAATTCTCACAACAAAAAGCCAGGAGTGT |
| 41 | AD-NnJAZ1 -F | CAGATTACGCTCATATGATGGCAAGTTCGCCGGATTCG |
| 42 | AD-NnJAZ1 -R | TGCTTGGGTGGAATTCCTATAGTTGGAAGTTAAATTGCTG |
| 43 | BD-NnWRKY70b-F | CATGGAGGCCGAATTCGGCGAGAGCAGGAAGTCTC |
| 44 | BD-NnWRKY70b-R | GCAGGTCGACGGATCCTTGGGGGGGCTTTGATATATC |
| 45 | B1-WRKY53b-F | ACAAGTTTGTACAAAAAAGCAGGCTTAATGGAGAACGCCGGGGAATG |
| 46 | B4-WRKY53b-R | ACAACTTTGTATAGAAAAGTTGGGTTCACAACAAAAAGCCAGGAGTGT |
| 47 | B1-WRKY70a-F | ACAAGTTTGTACAAAAAAGCAGGCTTAATGGGGACAGTGGAATCTCC |
| 48 | B4-WRKY70a-R | ACAACTTTGTATAGAAAAGTTGGGTTTACATTGAAACCTGAAAGATGTC |
| 49 | B1-WRKY70b-F | ACAAGTTTGTACAAAAAAGCAGGCTTAATGGACACAACTGTTGAATCTCC |
| 50 | B4-WRKY70b-R | ACAACTTTGTATAGAAAAGTTGGGTTTAAACCGGAAAAAAGTCATCATC |
| 51 | B1-JAZ1-F | ACAAGTTTGTACAAAAAAGCAGGCTTAATGGCAAGTTCGCCGGATTCG |
| 52 | B4-JAZ1-R | ACAACTTTGTATAGAAAAGTTGGGTCTATAGTTGGAAGTTAAATTGCTG |
| 53 | B3-WRKY70b-F | ACAACTTTGTATAATAAAGTTGGAATGGACACAACTGTTGAATCTCC |
| 54 | B2-WRKY70b-R | ACCACTTTGTACAAGAAAGCTGGGTAACCGGAAAAAAGTCATCATCATC |
| 55 | pTYDC1Y1_F | TTGAATTCGAGCTCGGTACCCGGGCAATCAAATCACGAACC |
| 56 | pTYDC1Y1_R | GAGCACATGCCTCGAGGCTGGAGATGGAGATGGAGATG |
| 57 | pNCS1Y1_F | TTGAATTCGAGCTCGGTACCACACGCTATCACAAACCTACCT |
| 58 | pNCS1Y1_R | GAGCACATGCCTCGAGCTTTTTCTTCGTTGGAAGGATGCA |
| 59 | p6OMT1Y1_F | TTGAATTCGAGCTCGGTACCGGGACAAGGCTAACAGCAATCT |
| 60 | p6OMT1Y1_R | GAGCACATGCCTCGAGTCTCCTCTCTATTCTATGCCCCT |
| 61 | p7OMT1Y1_F | TTGAATTCGAGCTCGGTACCCTGACTGAAGCCGAACTCTAT |
| 62 | p7OMT1Y1_R | GAGCACATGCCTCGAGTCTCCTCTCTATTCTATGCCCCT |
| 63 | TYDCF2 | CATTTTCGGCCGCAGCGTATAAACATTAGGTGGTTCACATTGC |
| 64 | TYDCF3 | CAAGGCAGTAGATAGCATTTCCACTCCAATCTATTGATTGGGTTGG |
| 65 | TYDCR1 | GCAATGTGAACCACCTAATGTTTATACGCTGCGGCCGAAAATG |
| 66 | TYDCR2 | CCAACCCAATCAATAGATTGGAGTGGAAATGCTATCTACTGCCTTG |
| 67 | 6OMTR1 | GTGATTCATTAGCGATGAATTGCTGATGGCGCAAGAGATCGTAG |
| 68 | 6OMTF2 | CTACGATCTCTTGCGCCATCAGCAATTCATCGCTAATGAATCAC |
| 69 | 6OMTR2 | GTAGACGTAGTAGTACATTCCTTCCACGCGCTAGAGCATGAAC |
| 70 | 6OMTF3 | GTTCATGCTCTAGCGCGTGGAAGGAATGTACTACTACGTCTAC |
| 71 | 7OMTR1 | GTTCGGGTCTTAATCGATTCAGCCGATCCTGACTCAATAATAATAG |
| 72 | 7OMTF2 | CTATTATTATTGAGTCAGGATCGGCTGAATCGATTAAGACCCGAAC |
| 73 | 7OMTR2 | CATTACTTCCCGTTGTAGTCGAGTGGGTAAGACTGGAAATTGG |
| 74 | 7OMTF3 | CCAATTTCCAGTCTTACCCACTCGACTACAACGGGAAGTAATG |
| 75 | CYP80GR1 | CGTCTGTCTATTGTATCAATTTGTCTTGGTCTTGCGATGGGCTAC |
| 76 | CYP80GF2 | GTAGCCCATCGCAAGACCAAGACAAATTGATACAATAGACAGACG |
| 77 | CYP719R2 | CTGGGGGTTTAAGCTATGCTGGAGCCGCCTTGAAGAAGAAG |
| 78 | CYP719F3 | CTTCTTCTTCAAGGCGGCTCCAGCATAGCTTAAACCCCCAG |
| 79 | CYP719R3 | GTGGGTTTTACTTTTAACTAAGGTTTCCATTGGTTTTGATTTTGAAGACC |
| 80 | CYP719F4 | GGTCTTCAAAATCAAAACCAATGGAAACCTTAGTTAAAAGTAAAACCCAC |
| 81 | ABACYP80GF1 | TTGAATTCGAGCTCGGTACC GTGGATCCATAAACTATATGAGTG |
| 82 | ABACYP80GR2 | GAGCACATGCCTCGAG TAGAGTCTATAGAGAAGATGTAGTAC |
| 83 | ABACYP719F1 | TTGAATTCGAGCTCGGTACC CGTATACCCATACTACAAGGTC |
| 84 | ABACYP719F2 | TTGAATTCGAGCTCGGTACC TCACCCTCCATCATAAAGCAAC |
| 85 | ABACYP719R4 | GAGCACATGCCTCGAG CCAATGCATCTAACTGATCAATC |
| 86 | ABACYP719R5 | GAGCACATGCCTCGAG CTCCGTTAACAGTCTCTCTCTC |

**Table S2** WRKY transcription factors involved in plant secondary metabolite biosynthesis.

| **Protein Name** | **Species** | **Genbank accession numbers** |
| --- | --- | --- |
| AaWRKY1 | *Artemisia annua* | FJ390842 |
| AaGSW1 | *Artemisia annua* | KX465128 |
| AtWRKY1 | *Arabidopsis thaliana* | AF442389 |
| AtWRKY12 | *Arabidopsis thaliana* | AF404857 |
| AtWRKY23 | *Arabidopsis thaliana* | AY052647 |
| AtWRKY33 | *Arabidopsis thaliana* | AK226301 |
| AtWRKY44 | *Arabidopsis thaliana* | NM_129282 |
| AtWRKY70 | *Arabidopsis thaliana* | NP_191199.1 |
| CrWRKY1 | *Catharanthus roseus* | HQ646368 |
| CjWRKY1 | *Coptis japonica* | AB267401 |
| GaWRKY1 | *Gossypium arboreum* | AY507929 |
| HbWRKY1 | *Hevea brasiliensis* | JF742559 |
| HbWRKY41 | *Hevea brasiliensis* | GU372969 |
| MdWRKY100577 | *Medicago truncatula* | EU526033 |
| MdWRKY100630 | *Medicago truncatula* | EU526034 |
| MdWRKY108715 | *Medicago truncatula* | EU526035 |
| MdWRKY109669 | *Medicago truncatula* | EU526036 |
| OsWRKY13 | *Oryza sativa* | EF143611 |
| OsWRKY45 | *Oryza sativa* | AK066255 |
| OsWRKY53 | *Oryza sativa* | AB190436 |
| OsWRKY76 | *Oryza sativa* | AK068337 |
| OsWRKY89 | *Oryza sativa* | AY781112 |
| PqWRKY1 | *Panax quinquefolius* | JF508376 |
| PsWRKY1 | *Papaver somniferum* | JQ775582 |
| SlWRKY73 | *Solanum lycopersicum* | NM_001247873 |
| TcWRKY1 | *Taxus chinensis* | JQ250831 |
| VvWRKY2 | *Vitis vinifera* | AY596466 |
| WsWRKY1 | *Withania somnifera* | GR923578 |
| NnWRKY70a | *Nelumbo nucifera* | OL469000 |
| NnWRKY70b | *Nelumbo nucifera* | OL469001 |
| NnWRKY53b | *Nelumbo nucifera* | OL468999 |

**Table S3** Yeast two-hybrid library screening obtained proteins putatively interacted with NnWRKY70b.

| **No** | **NCBI ID Matching List** | **Predicted Function** |
| --- | --- | --- |
| 1 | XP_010260275.1 | auxin-repressed 12.5 kDa protein-like isoform X1 |
| 2 | XP_010266466.1 | probable WRKY transcription factor 41 [NnWRKY53b in our previous report (Li et al., 2019)] |
| 3 | XP_010267058.1 | PITH domain-containing protein 1 |
| 4 | XP_010274087.1 | HNH nucleases |
| 5 | XP_010251469.1 | protein TIFY 10A-like (NnJAZ1 in this research) |
| 6 | XP_010248363.1 | ras-related protein RIC2 |
| 7 | XP_010273935.1 | 40S ribosomal protein S3-3-like |
| 8 | XP_010274017.1 | carboxylesterase 1-like |
| 9 | XP_010271476.1 | fructose-bisphosphate aldolase 1 |
| 10 | XP_010250427.1 | glyceraldehyde-3-phosphate dehydrogenase A, chloroplastic |
| 11 | XP_010262976.1 | NDR1/HIN1-like protein 12 |
| 12 | XP_010243512.1 | NAC domain-containing protein 2-like |
| 13 | XP_010268312.1 | 40S ribosomal protein S24-1 |
| 14 | XP_010262413.1 | multiple organellar RNA editing factor 2, chloroplastic |
| 15 | XP_010270134.1 | glycerophosphodiester phosphodiesterase GDPDL4 |
| 16 | XP_010265138.1 | ribulose bisphosphate carboxylase small chain, chloroplastic-like |
| 17 | XP_010262214.1 | Mitochondrial ATP synthase g subunit |
| 18 | XP_010256118.1 | proteasome subunit beta type-2-B |
| 19 | XP_010251601.1 | reticulon-like protein B2 |
| 20 | XP_010267886.1 | 2S sulfur-rich seed storage protein 2-like |
| 21 | XP_010262323.1 | glutelin type-B 5-like |
| 22 | XP_010275653.1 | probable mediator of RNA polymerase II transcription subunit 36b |
| 23 | XP_010268436.1 | uncharacterized protein LOC104605392 |
| 24 | XP_010253266.1 | actin-depolymerizing factor 2 |
| 25 | XP_010247657.1 | 60S acidic ribosomal protein P0-like |
| 26 | XP_010270953.1 | S-adenosylmethionine synthase 5 |
| 27 | XP_010276554.1 | uncharacterized protein LOC104611264 |
| 28 | XP_010248518.1 | uncharacterized protein LOC104591415 |
| 29 | XP_010263125.1 | ATP synthase delta chain, chloroplastic |
| 30 | XP_010244725.1 | ACT domain-containing protein ACR12-like |
| 31 | XP_010266914.1 | plastocyanin B'/B'' |
| 32 | XP_010256830.1 | protein plastid transcriptionally active 16, chloroplastic |


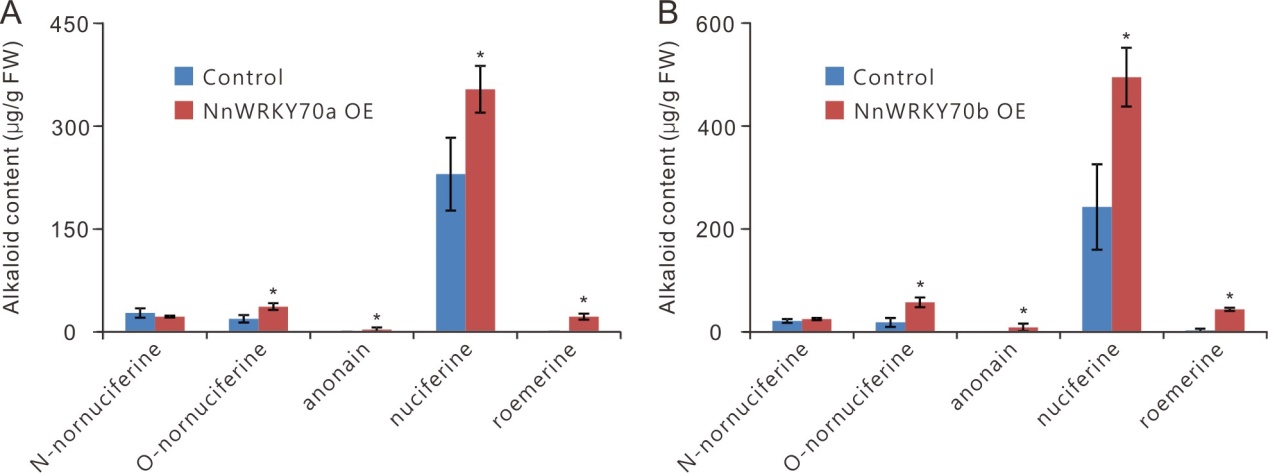


**Figure S1** Changes of benzylisoquinoline alkaloids in lotus leaves by the over expression of NnWRKY70a (A) and NnWRKY70b (B). Asterisks (*) represents significant differences at *P* < 0.05.


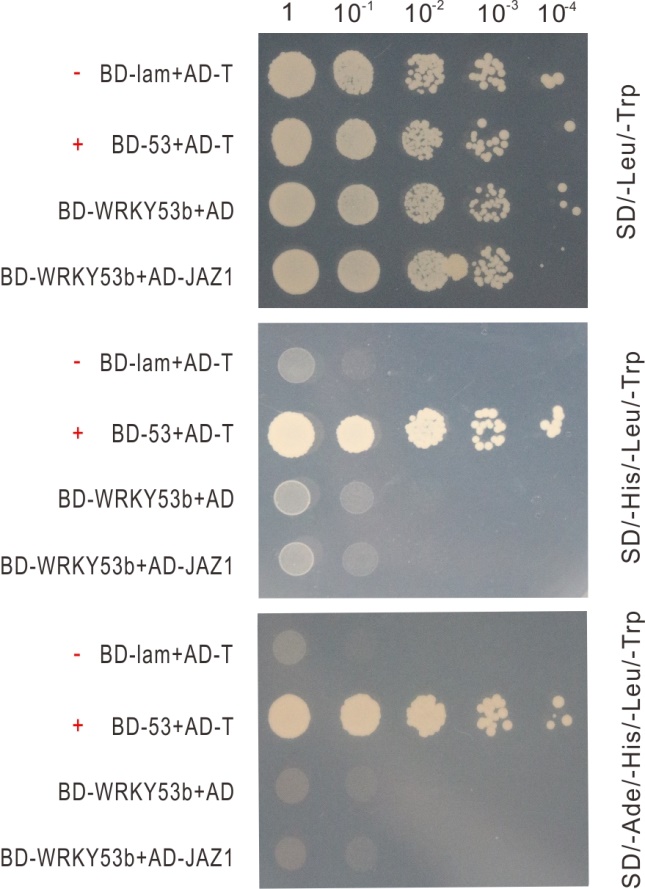


**Figure S2** Yeast two-hybrid (Y2H) assay checking the interaction between NnWRKY53b and NnJAZ1. Y2H experiments were conducted by following the Matchmaker® Gold Yeast Two-Hybrid System User Manual. BD, bait protein expression vector pGBKT7; AD, prey protein expression vector pGADT7; positive control, pGBKT7-53+ pGADT7-T; negative control, pGBKT7-lam+ pGADT7-T.


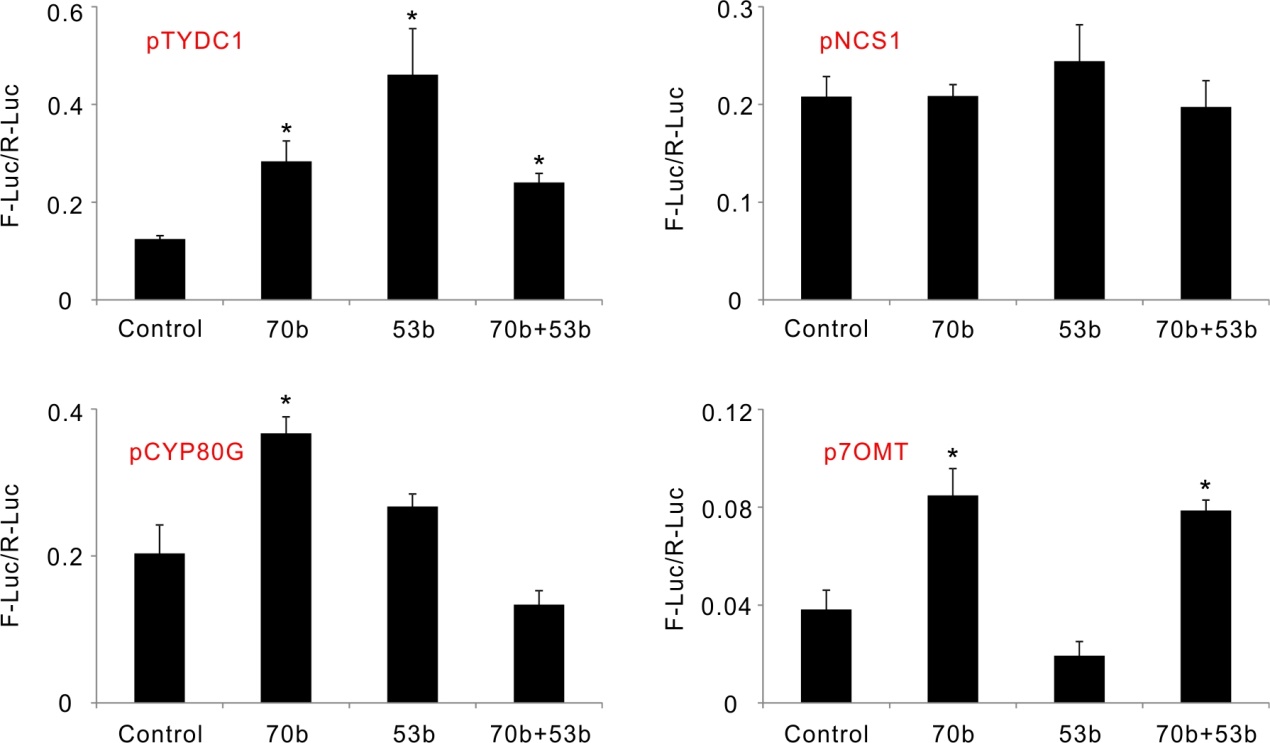


**Figure S3** Dual luciferase assays assessing the effect of NnWRKY70b (70b), NnWRKY53b (53b), and NnWRKY70b+NnWRKY53b (70b+53b) on activation BIA pathway gene promoters.
